# Are parents’ geographical origin associated with their evaluation of child and adolescent mental health services? Results from a national survey in Norway

**DOI:** 10.1007/s00787-020-01590-9

**Published:** 2020-07-02

**Authors:** Oyvind Bjertnaes, Hilde Hestad Iversen, Kjersti Eeg Skudal, Warsame Abdullahi Ali, Ketil Hanssen-Bauer

**Affiliations:** 1 Department for Health Services Research, Norwegian Institute of Public Health, Oslo, Norway; 2 Division of Mental Health Services, Akershus University Hospital, Lørenskog, Norway; 3 Clinic for Health Service Research and Psychiatry, Institute of Clinical Medicine, University of Oslo, Oslo, Norway

**Keywords:** Parent satisfaction, Quality, Child and adolescent mental health services, Survey

## Abstract

The objective was to assess the association between parents’ geographical origin and their evaluation of outpatient child and adolescent mental health services (CAMHS). Data were collected in a national parent’s experience survey of all outpatient CAMHS in Norway in 2017. Following exclusions, 16,143 parents were part of the study, of which 5932 responded (36.1%). Diagnosis and global psychosocial functional level were collected from the National Patient Register. Multilevel regression was used to assess the association between parents’ geographical origin and parent evaluation of the outpatient CAMHS on ten indicators. Sentiment and content analysis was conducted on open-ended comments from parents. The estimated regression coefficients showed that parents born in Eastern Europe scored the services significantly poorer than parents born in Norway on outcome of treatment (− 7.73, *p* < 0.01), general satisfaction (− 5.53, *p* < 0.05), ease of getting in contact with health personnel outside of scheduled appointments (− 17.04, *p* < 0.001), and knowledge of the services that the child has received at the service (− 10.63, *p* < 0.001). Parents born in Asia/Africa/South America scored the services similar as Norwegian parents on eight of ten indicators, better on one (waiting time) and poorer on one (ease of getting in contact). Sentiment analysis showed that 54% of the comments from parents born in Eastern Europe were negative, compared to 42% for the Norwegian group and 36% for Asia/Africa/South America. The parents’ evaluation of the outpatient CAMHS were partly associated with their geographical origin, with parents born in Eastern Europe reporting poorer experiences than parents born in Norway.

## Introduction

Patient centredness is an important quality component in itself [1], and positive patient experiences are associated with better adherence to treatment recommendations, better patient safety, better effectiveness, and less health care utilization [2, 3]. Patient satisfaction has been used as an outcome indicator in child and adolescent mental health services (CAMHS) for a long time [4], but the patient voice is often represented or supplemented by parent satisfaction [5–10], and even the clinician perspective [11]. While such perspectives are crucial in quality measurement, health system performance also involves assessment of equity or the fairness of the distribution of health care and quality across populations [1]. With the huge amount of people living in other countries than the one they were born in, an important equity assessment is the potential quality gap between the majority population and immigrants. In Norway, 17% of the population in 2018 were either first-generation immigrants or Norwegian-born with immigrant parents.

A number of studies have been conducted to explore patient experiences in immigrant groups or by ethnicity/race, in a range of settings [12–22], and most have found poorer experiences in minority groups than in the majority population. However, the knowledge base on the association between race/ethnicity and patient experiences in CAMHS is scarce. A few studies have indirectly assessed the topic in general predictor studies [6–8], using responses from children themselves or parents/proxies for children. One US study found that youth satisfaction was higher for patients with Caucasian ethnicity than others, but not for parents [7]. A Norwegian study found small differences when comparing parent experiences between Norwegians and other ethnic backgrounds/languages [6], in line with an Australian study that included culturally and linguistically diverse status and Aboriginal and Torres Strait Islander status as predictors of patient satisfaction [8]. We also found a relevant study with non-significant differences between the minority and majority population, but with a very small and statistically uncertain ethnicity sample [10]. Furthermore, predictor studies in this field without ethnicity or similar variables were also identified [9]. The small amount of studies, combined with the methodological weaknesses and variation in results, warrants more research into the level of parent experiences in different ethnic groups.

The Norwegian Institute of Public Health (NIPH) is responsible for national patient experience surveys in Norway. In 2017, the NIPH conducted a national parent experience survey with outpatient CAMHS. The survey included a number of items and indicators about parent experiences with the outpatient CAMHS, background questions about the parent respondents including geographical origin, and clinical data about the patient from the National Patient Register. The main objective of the current study was to assess the association between parents’ geographical origin and their experiences with the outpatient clinics, adjusting for other predictors at the individual level, and using multilevel analysis to adjust for the health care level. The knowledge base from this setting is scarce, but based on the general literature [12–22], we expected minority parents to report poorer experiences than parents born in Norway. Based on recent Norwegian findings on patient experiences with general practitioners [23], we expected poorer experiences for parents born in Asia, Africa, South America, and Eastern Europe.

## Methods

The Norwegian Directorate of Health commissioned the survey from the National Institute of Public Health (NIPH), as basis for national quality indicators, quality-based financing, and measurements related to the implementation of clinical pathways.

### Data collection

The patient sample consisted of children and adolescents below 16 years receiving outpatient mental health services from CAMHS in 2017 in Norway. At that time, there were 99 outpatient CAMHS in Norway. For each outpatient, CAMHS 400 patients were randomly selected from the national patient register or all patients for services with less than 400 patients in 2017. Patients with less than four consultations in 2017 were excluded, except patients first time registered at the service between 1st of November and 31st of December. We excluded deceased patients. Patients were only included once, and we selected the last service if patients had attended more than one in 2017. The services were responsible for excluding patients if parents were unaware of the child’s contact with the service, or if the patient was registered with the code “secret address”. Services not conducting and formally confirming the exclusion procedure were excluded from the survey (*n* = 12), and we excluded services not signing the formal agreement with the NIPH (*n* = 6) and one service with a systematic error in the patient list. Following these exclusions, we included 80 outpatient services in the survey. We mailed the questionnaire to the parents of patients, with up to two reminders to non-respondents. The first request only included an electronic response option, while the reminders included both a pen-and-paper questionnaire and an electronic response option. The corrected patient sample consisted of 16,413 patients, of which 5932 parents responded (36.1%). The 80 outpatient CAMHS had on average 74 responses, but wide variation in the number of responses (range 1–181), and with seven CAMHS having ten or fewer responses.

For respondents and non-respondents not actively reserving themselves from the survey, we gathered socio-demographic variables and clinical variables from the National patient register. This included patient age, gender, referral reason(s), diagnoses (including global psychosocial functional level), and duration of contact with the clinic and clinic identificator.

### Questionnaire

The questionnaire comprised 39 closed-ended items including parent evaluation questions and background questions, building on a previously validated Norwegian questionnaire [24]. We updated the questionnaire according to the newest standards for questionnaires in the NIPH, for instance concerning layout and the use of non-applicable response options. Furthermore, we included four items of special relevance for the Norwegian Directorate of Healths’ national work related to clinical pathways. Geographical origin of the parent was included as a background variable, with the following response categories: (i) Norway; (ii) Asia/Africa/South America; (iii) Eastern Europe; (iv) Nordic, West Europe, North America, and Oceania. The geographical origin variable is one of the standards used by Norwegian Statistics, and was not amended in this study.

Most parent evaluation items had a five-point response format ranging from 1 (‘not at all’) to 5 (‘to a very large extent’). A total of 20 items related to parent evaluation were basis for four scales: relationship and influence (13 items), perceived benefit of treatment (two items), changes in child functioning since treatment start (three items), and general satisfaction (two items). In addition, we used six single items as indicators in this study: waiting time before being offered help at the clinic, the occurrence of offensive episodes towards the child at the clinic, the occurrence of offensive episodes towards the parent at the clinic, assessment of the number of consultations at the clinic, ease of getting in contact with health personnel outside of scheduled appointments, and knowledge of the services that the child has received at the clinic. All scales and items were linearly transformed to a 0–100 scale [21], where 100 is the best possible score.

### Diagnosis data

We included the diagnoses for each child at CAMHS. They use a Norwegian version of a multiaxial system of six axes based on the International Classification of Diseases (ICD)-10 [25, 26]. Axis 6 refers to the child’s level of global psychosocial functioning as measured by the Children’s global assessment scale (CGAS) [27], rated from 1 (Needs constant supervision) to 100 (superior functioning in all areas (at home, at school, and with peers)). CGAS was kept as a separate variable.

### Statistical analysis

Descriptives on background variables for the four geographical origin groups were computed, with Chi-square tests for categorical variables and one-way ANOVA for continuous variables. Bivariate associations between parents’ geographical origin and the ten indicators on outpatient evaluation were tested by means of one-way ANOVA. For one-way ANOVA, we first used the standard *F* test, testing the hypothesis that all means are equal versus the alternative that at least one mean is different from the rest. We then applied the Bonferroni method to correct for multiple testing. To account for the clustering of patients within health care units and adjust for potential confounders, we conducted multilevel regression analysis for each of the ten indicators. The health care enterprises were included as random intercepts (18 enterprises with n ranging from 44 to 672), and we used the following independent variables at the individual level: patient age, gender, diagnosis, functional level, duration of contact with the clinic, parents’ education level, civil status (married/cohabitant or not), type of respondent (mother, father, both, other), parent age, and geographical origin. The latter was included as three dummy variables, with Norway as reference category. The geographical origin regression estimate in each multilevel model was of primary interest (the unstandardized coefficient), which can be interpreted as the difference in parent evaluation (on the 0–100 scale) between a specific geographical origin group and Norway after adjustments.

### Analysis of free-text comments

The questionnaire included an open space where participants were invited to elaborate further on their experiences with the CAMHS. Parents from the groups Nordic, West Europe, North America, and Oceania and Norway were more likely to leave comments than parents born in Asia/Africa/South America and Eastern Europe. Forty-seven percent of the respondents born in the group Nordic, West Europe, North America, and Oceania left an open-ended comment, and 43% of parents born in Norway. The percentages for parents born in Asia/Africa/South America and Easter Europe were 31% and 33%, respectively. Two researchers independently coded open-ended comments from a group of randomly selected Norwegian-born parents (99 of 2335 comments) and all comments from parents born in other countries: 76 from Asia/Africa/South America, 50 comments from parents born in Eastern Europe, and 112 from Scandinavia, West Europe/North America/Oceania. Coding conflicts were resolved through discussion.

Comments from each of the four geographical origin groups were analyzed separately using thematic analysis, employing an inductive approach where coding and theme development were driven by the content of the comments. To structure the data, responses were sorted into first-order codes based on whether the comment was (1) positive; (2) negative; (3) both positive and negative, or (4) neutral or miscellaneous. Neutral comments that did not address the CAMHS or give any specific evaluation of the healthcare quality were not included in further analyses. Next, second-order codes were applied to each of the four individual data sets to identify main themes and subthemes. The codes were inductively driven, and the coding sheets were different for each of the data sets. Comments were given as many codes as were appropriate to cover the content of the comment.

## Results

The geographical origin groups differed significantly on most background variables, with the exception of childs’ gender, global psychosocial functional level and duration of contact with the clinic (Table 1). Patients with parents from Asia/Africa/South America and from Eastern Europe were younger than patients with parents from the other groups (*p* < 0.001), with the former about 1.3 years younger than the Norwegian group (10.1 versus 11.4). The groups also differed significantly regarding type of diagnosis (*p* < 0.001): for instance, 41.2% of the group Asia/Africa/South America did not have a mental/behavioural/psychosocial disorder (compared to around 30–32% in the other groups), while 17% of the group Eastern Europe had a developmental disorder (compared to 7–12% in the other groups). Education level also differed between the groups (*p* < 0.001), with the group Asia/Africa/South America having the lowest education level (20.7% only had primary school, compared to 3–4% in the other groups). Norwegians had the lowest percentage of married/cohabitant parents (56.1% versus 62–64%), while the group Asia/Africa/South America had the lowest percentage of mother as respondent (58.8% versus 70–78%). Finally, respondent age differed significantly between the groups (*p* < 0.001), with Asia/Africa/South America and Eastern Europe having the youngest respondents.

**Table 1:** Descriptives for patients and parents by geographical origin of parent (*n* = 5882)

	Norway (*n* = 5248)	Asia, Africa, South America (*n* = 245)	Eastern Europe (*n* = 153)	Nordic, West Europe, North America, Oceania (*n* = 236)	*P* value^b^
Child’s age, mean (sd)	11.4 (3.3)	10.1 (3.8)	10.6 (3.7)	11.4 (3.5)	< 0.001
Child’s gender, *n* (%)					0.159
Boys	3080 (58.7)	155 (63.3)	101 (66.0)	138 (58.5)	
Girls	2168 (41.3)	90 (36.7)	52 (34.0)	98 (41.5)	
Global psychosocial functional level child,^a^ mean (sd)	56.0 (9.5)	54.7 (12.3)	55.1 (11.1)	56.6 (11.2)	0.425
Diagnosis child (axes 1, 2, 3, and 5), *n* (%)					< 0.001
Hyperkinetic or conduct disorders	1179 (22.5)	39 (15.9)	21 (13.7)	47 (19.9)	
Emotional disorders	1072 (20.4)	35 (14.3)	29 (19.0)	53 (22.5)	
Developmental disorders	359 (6.8)	29 (11.8)	26 (17.0)	18 (7.6)	
Psychosocial problems only	484 (9.2)	28 (11.4)	13 (8.5)	24 (10.2)	
Other mental or behavioural disorders	374 (7.1)	9 (3.7)	13 (8.5)	15 (6.4)	
No mental/behavioural/psychosocial disorders	1714 (32.7)	101 (41.2)	48 (31.4)	71 (30.1)	
Lack diagnosis	66 (1.3)	4 (1.6)	3 (2.0)	8 (3.4)	
Duration of contact with service, *n* (%)					0.529
Less than 3 months	592 (11.3)	31 (12.7)	18 (11.8)	25 (10.6)	
3–12 months	2463 (46.9)	120 (49.0)	82 (53.6)	118 (50.0)	
More than a year	2193 (41.8)	94 (38.4)	53 (34.6)	93 (39.4)	
Highest education responding parent, *n* (%)					< 0.001
Primary school	197 (3.8)	49 (20.7)	5 (3.3)	8 (3.4)	
Secondary school	1694 (32.4)	84 (35.4)	53 (34.6)	47 (19.9)	
University/college degree (0–4 years)	1651 (31.6)	62 (26.2)	46 (30.1)	74 (31.4)	
University/college degree (4+)	1680 (32.2)	42 (17.7)	49 (32.0)	107 (45.3)	
Parent married/cohabitant, *n* (%)					< 0.01
Yes	2933 (56.1)	157 (64.1)	96 (63.2)	147 (62.3)	
No	2294 (43.9)	88 (35.9)	56 (36.8)	89 (37.7)	
Respondent type, *n* (%)					< 0.001
Mother	3648 (69.6)	144 (58.8)	109 (71.2)	183 (77.5)	
Father	705 (13.5)	38 (15.5)	12 (7.8)	21 (8.9)	
Both parents	700 (13.4)	58 (23.7)	27 (17.6)	29 (12.3)	
Other	188 (3.6)	5 (2.0)	5 (3.3)	3 (1.3)	
Respondent age, *n* (%)					< 0.001
Below 40 years	1527 (30.4)	92 (40.4)	70 (49.3)	65 (28.3)	
40–46 years	1968 (39.2)	87 (38.2)	48 (33.8)	89 (38.7)	
47 years or older	1530 (30.4)	49 (21.5)	24 (16.9)	76 (33.0)	

^a^ Global psychosocial functional level was measured with the use of Children’s global assessment scale (CGAS) scored 1–100 by clinicians, where 1 represents “Needs constant supervision” and 100 represents “Superior functioning in all areas (at home, at school, and with peers)

^b^ Chi-square test for categorical variables; one-way ANOVA for continuous variables

In bivariate analysis, geographical origin of parents was significantly associated with four of ten indicators (Table 2): outcome of treatment for child and parent (*p* < 0.05), waiting time before offered help at the service (*p* < 0.01), ease of getting in contact with health personnel outside of scheduled appointments (*p* < 0.001), and knowledge of the services the child has received at the service (*p* < 0.001). Following Bonferroni corrections for multiple testing, Eastern Europe was significantly different from Norwegian-born parents on three indicators (outcome, ease of getting in contact, and knowledge of the clinic), while Asia/Africa/South America were significantly different from Norwegian-born parents on two indicators (waiting time and ease of getting in contact).

**Table 2:** Parent evaluation of outpatient mental health services by geographical origin of parent (*n* = 5882)

	Norway (*n* = 5248)	Asia, Africa, South America (*n* = 245)	Eastern Europe (*n* = 153)	Nordic, West Europe, North America, Oceania (*n* = 236)	*P* value^b^
Scales^a^					
Experiences with health personnel (13 items)	68.2	69.4	67.0	68.1	0.743
Outcome of treatment for child and parent (2 items)	59.4	58.4	52.2	57.5	0.026
Changes in child health/functioning compared to prior to treatment (3 items)	57.8	57.8	59.1	58.5	0.920
General satisfaction (2 items)	72.8	72.2	67.8	70.1	0.061
Single items^a^					
Waiting time before being offered help at the clinic	61.7	68.0	62.3	60.0	0.002
Offensive episodes child	96.8	96.1	96.6	97.7	0.679
Offensive episodes parent	92.9	93.9	94.7	93.4	0.600
Assessment of the number of consultations at the clinic	68.8	72.5	66.9	69.2	0.642
Ease of getting in contact with health personnel outside of scheduled appointments	50.7	36.1	34.1	49.7	< 0.001
Knowledge of the services the child has received at the clinic	70.0	66.1	59.7	68.9	< 0.001

^a^ All scales and items scored 0–100, where 100 represents the best possible experiences

^b^ One-way ANOVA

Multilevel regressions with parents born in Norway versus parents from all other groups showed that four of ten regressions were significant: outcome of treatment for child and parent (estimate: 3.22, *p* < 0.05), general satisfaction (estimate: 2.78, *p* < 0.05), ease of getting in contact with health personnel outside of scheduled appointments (estimate: 10.34, *p* < 0.001), and knowledge of the services that the child has received at the service (estimate: 4.39, *p* < 0.001). In multilevel regressions comparing parents born in Norway and each of the other groups, parents born in Eastern Europe scored the services significantly poorer than parents born in Norway on four of ten indicators (Table 3): outcome of treatment (estimate: − 7.73, *p* < 0.01), general satisfaction (estimate: − 5.53, *p* < 0.05), ease of getting in contact with health personnel outside of scheduled appointments (estimate: − 17.04, *p* < 0.001), and knowledge of the services that the child has received at the service (estimate: − 10.63, *p* < 0.001). Parents born in Asia/Africa/South America scored the services significantly poorer than parents born in Norway on ease of getting in contact with health personnel outside of scheduled appointments (estimate: − 15.57, *p* < 0.001), but better on perceived waiting time (estimate: 5.40, *p* < 0.01). None of the differences between Norway and the group Nordic, West Europe, North America, and Oceania were significant, and half of the estimates were positive and half negative.

**Table 3:** Results of multilevel regressions of the association between geographical origin and parent evaluation, with Norway as reference category

	Asia, Africa, South America (vs Norway)		Eastern Europe (vs Norway)		Nordic, West Europe, North America, Oceania (vs Norway)	
	Estimate	*P* value	Estimate	*P* value	Estimate	*P* value
Scales						
Experiences with health personnel (13 items)	− 0.158	0.912	− 2.066	0.237	0.344	0.806
Outcome of treatment for child and parent (2 items)	− 2.184	0.281	− 7.728	0.002	− 1.512	0.430
Changes in child health/functioning compared to prior to treatment (3 items)	− 0.910	0.619	1.531	0.492	0.960	0.596
General satisfaction (2 items)	− 1.597	0.371	− 5.527	0.012	− 2.184	0.216
Single items						
Waiting time before being offered help at the clinic	5.40	0.002	0.498	0.814	− 0.664	0.700
Offensive episodes child	− 1.440	0.132	− 0.348	0.764	1.159	0.210
Offensive episodes parent	0.314	0.818	1.773	0.288	0.781	0.556
Assessment of the number of consultations at the clinic	3.059	0.337	− 2.118	0.583	1.213	0.698
Ease of getting in contact with health personnel outside of scheduled appointments	− 15.565	< 0.001	− 17.042	< 0.001	− 0.529	0.798
Knowledge of the services the child has received at the clinic	− 3.111	0.077	− 10.632	< 0.001	− 1.674	0.344

Adjusted for patient age, gender, diagnosis and functional level, duration of contact with the clinic, parents’ education level, civil status (married/cohabitant or not), type of respondent (mother, father, both, other), parent age, and the health care enterprise level. Estimate is the unstandardized regression coefficient, and can be interpreted as the difference (on the 0–100 scale) from parents born in Norway after adjustments

### Results of free-text analyses

The first-order coding showed that overall, more negative than positive comments were made by all groups, except for parents born in Asia/Africa or South America (Table 4). The results for the group Nordic, West Europe, North America, and Oceania were similar to the results for the parents born in Norway. 54% of the comments made by parents from Eastern Europe were categorized as negative, compared to 49% in the western group, 42% in the Norwegian group, and 36% in the group born in Asia/Africa/South America (Table 4). Results from the second-order coding showed that the largest single theme commented on was organization and availability of the mental health services. Within this theme, respondents often described clinician continuity, waiting time for consultations, and timeliness to consultations. The theme receiving the second largest volume of comments was outcome of treatment and the treatment itself, and addressed the parents perceived general outcome for the child, how the treatment affected the child’s function in and outside the family and reflections regarding the chosen treatment and focus in the consultations. Communication and information included the child’s and parents’ interactions and communication with health professionals, and health professionals’ skills related to listening, sharing decisions and giving information/education. The most frequently mentioned topics were similar in all groups, and no significant differences between the groups were identified in the second-order coding.

**Table 4:** Sentiment analysis of open-ended comments by geographical origin (%)

	Norway (*n* = 99)	Asia, Africa, South America (*n* = 76)	Eastern Europe (*n* = 50)	Scandinavia, West Europe, North America, Oceania (*n* = 111)
Positive	19	37	14	16
Negative	42	36	54	49
Both positive and negative	24	12	16	23
Neutral	14	16	16	12

## Discussion

The parents’ evaluation of the outpatient CAMHS was partly associated with their geographical origin, with parents born in Eastern Europe reporting poorer experiences than parents born in Norway. Parents born in Asia/Africa/South America had rather similar levels of experiences as parents born in Norway, but their comments were much more positive than parents from other groups.

Based on the general literature and recent Norwegian findings on patient experiences with general practitioners [12–23], we expected minority parents to report poorer experiences than parents born in Norway. This study supported the hypothesis for parents born in Eastern Europe: four of ten parent experience indicators were significantly poorer than in the Norwegian group, and the percentage of negative parent comments were higher than for Norwegians (54% versus 42%). Parents from Eastern Europe were less satisfied with the CAMHS and reported poorer outcome of treatment, in addition to access barriers between consultations and poorer knowledge of the CAMHS. Previous qualitative studies conducted in Norway among polish immigrants point to additional general challenges for the parent group in our study [28, 29]. Semi-structured interviews with polish immigrants and thematic analysis were performed to identify barriers and facilitators related to the access to health services in general [28], and more specifically to interpreter services [29]. The most common barriers in the former study were insufficient language skills, communication problems, and lack of knowledge about navigating in the Norwegian health care system, and the latter found that access to interpretation services was limited or denied. While parents from Eastern Europe in our study reported similar experience as parents born in Norway on several indicators, both knowledge of the services, access between scheduled appointments and perceived outcome of treatment and satisfaction were significantly lower. It seems like initiatives to increase these parents’ competence about CAMHS and to involve them more in consultations which would be a fruitful avenue for possible improvements in scores.

Parents born in Asia/Africa/South America reported similar experiences as parents born in Norway, except for indicators related to getting in contact with health personnel between appointments (more negative) and waiting time before being offered help at the service (more positive). This is positive and could indicate that CAMHS are responsive to the needs of these groups. Actually, sentiment analysis of open-ended comments of parents with origin in Asia/Africa/South America was much more positive then comments from other parents: almost 40% were positive compared to less than 20% in the other groups. Given that quantitative indicators were similar, the reasons for this are most likely related to the free-text comment approach itself, like more skepticism towards writing about negative experiences (including social desirability bias), a selection bias concerning who actually writes comments, or language barriers (more challenging to formulate criticism than short positive statements). All in all, the results shows that parents from Asia/Africa/South America are satisfied with the CAMHS, at least at the same level as Norwegian-born parents and at a higher level than parents from Eastern Europe. We observe that parents from Asia/Africa/South America report a better knowledge of the services which their children have received at the clinic than parents from Eastern Europe. This could indicate a better integration of parents from Asia/Africa/South America in the health care services received by their children. To increase the understanding of the differences, we suggest future in-depth qualitative work, including both the perspective of adolescents and parents. We also suggest to further study the positive finding about waiting time, especially by comparing subjective waiting time to objective waiting time to identify potential effects of different culture/expectations [15]. Our study found that a larger proportion of patients with parents from Asia/Africa/South America was classified with no mental/behavioural/psychosocial disorder (41.2% versus 31.4 for Eastern Europe), which could imply that the probability of uptake in mental health care varies with region of origin. This is also something that should be a topic for future research, given that resources are scarce and the need for services is large in this setting.

### Implications to policy and practice

A recent systematic review identified several barriers and facilitators for children and adolescents seeking and accessing professional help for mental health problems [30]. Previous research in Norway shows that children and adolescents with an immigrant background use specialist mental health care less than ethnic Norwegians, including immigrants from eastern parts of Europe, except Russia [31]. One report suggests that Poles often report travelling home to use health care services there instead of in Norway [32], but this seems less likely in this setting given the frequency of consultations in mental health care for young people. Combined with the results of the current study, one possible interpretation is that immigrant groups underutilize mental health services for children and adolescents, and that the actual users in some immigrant groups (Eastern Europe) receive poorer services than the majority population. This would be problematic for a range of reasons. Lack of access to effective treatment for young persons having health care needs might result in deteriorated mental health over time, with negative consequences for functioning in family, with friends, school, and work life. Poorer patient experiences with health services are negative in itself, but it also means less likelihood of the related positive outcomes, like better adherence to treatment recommendations, better patient safety, and better effectiveness [2, 3]. The scarcity of research on immigrants and patient experiences in this field means more research is needed, but together with findings on less access/use, our study underlines the importance of more focus on immigrants from Eastern Europe in policy and practice, the latter including cultural competence training of healthcare providers [33].

## Limitations

This study has several limitations. The geographic origin groups are very heterogenous, including, for instance, three continents for the category Asia/Africa/South America. This might mask important differences between parents from different continents or countries within continents. Future research should be conducted with more homogenous geographical origin groups, with samples for each group at least the size of the groups in the current study. Another limitation is the lack of data about geographic origin for non-respondents. The response rate is low and we are not able to rule out selection bias, e.g., if respondents selected to participate, because they are more satisfied/have better experiences than non-respondents. A previous follow-up study of non-respondents in Norway showed similar parent experience scores as original respondents [24], but that study did not specifically assess selection bias for different geographic origin groups. Since the questionnaire only was available in Norwegian, we expect non-respondents in the two relevant groups to be less proficient in Norwegian, and possibly to have even poorer experiences with the outpatient services than the responding parents. Future research should include geographical origin in the sample frame, boost sample size for each group to at least the level of the current study, and include translations of the questionnaire for the most common languages. Future research should also assess the effects of culture, expectations, and quality on the observed differences between parents born in Eastern Europe and parents born in Norway. Poorer parent-reported experiences might not be related to poorer quality, but could also be explained by differences in expectations and/or culture [15, 34]. Finally, while the robustness of modelling was supported by convergence of main results from different types of analysis (one-way ANOVA with and without Bonferroni corrections, and multilevel analysis with correction for both the provider level and background variables about the patients and the parents), the effect of non-measured variables like years of residence is unknown. Future research should identify and test potential predictors not included in the current study.

## Conclusions

The parents’ evaluation of the outpatient CAMHS was partly associated with their geographical origin, with parents born in Eastern Europe reporting poorer experiences than parents born in Norway. Future research assessing the effects of culture, expectations, and quality on the observed differences is warranted, as well as research with larger and more homogenous geographical origin groups.

## Data Availability

Data supporting the findings of this study are available from the corresponding author upon reasonable request, within the boundaries of juridical approvals and parent consent. Data are from a national survey of parent experiences with CAMHS in Norway, and the corresponding author may be contacted at the Department of Health Services Research, NIPH, Norway.
